# Ascertaining Out-of-Pocket Costs of Dementia Care: Feasibility Study of a Web-Based Weekly Survey

**DOI:** 10.2196/56878

**Published:** 2024-09-25

**Authors:** Walter D Dawson, Nora Mattek, Sarah Gothard, Jeffrey Kaye, Allison Lindauer

**Affiliations:** 1 Oregon Alzheimer’s Disease Research Center School of Medicine Oregon Health & Science University Portland, OR United States; 2 Global Brain Health Institute University of California San Francisco, CA United States; 3 Department of Neurology School of Medicine Oregon Health & Science University Portland, OR United States; 4 School of Nursing Oregon Health & Science University Portland, OR United States

**Keywords:** Alzheimer disease, caregiving, out-of-pocket costs, behavioral and psychological symptoms of dementia, technological interventions, clinical trials, dementias

## Abstract

**Background:**

Caring for a family member living with dementia is costly. A major contributor to care demands, and therefore to the costs, are the behavioral symptoms of dementia. Here, we examine the feasibility of ascertaining costs related to caregiving from weekly web-based surveys collected during a telehealth-based behavioral intervention study—Support via Technology: Living and Learning with Advancing Alzheimer Disease.

**Objective:**

This study aims to determine the feasibility and acceptability of using a web-based weekly survey to capture real-time data on out-of-pocket caregiving expenses and time commitments associated with dementia care. To examine relationships between behavioral symptoms, care partner reactivity, burden, and out-of-pocket dementia care costs.

**Methods:**

Feasibility was measured by accrual, retention, and data completion by participating care partners. Behavioral symptoms, care partner reactivity, and burden were collected before and after the intervention from 13 care partners. Weekly web-based surveys queried Support via Technology: Living and Learning with Advancing Alzheimer Disease care partners about their out-of-pocket costs associated with care-related activities. The surveys included questions on out-of-pocket costs care partners incurred from hospitalizations and emergency department use, primary care provider visits, use of paid in-home care or respite services, use of prescription medications, and use of over-the-counter medications. The surveys also queried the amount of time care partners devoted to these specific care–related activities.

**Results:**

Out-of-pocket costs of dementia care were collected via a web-based weekly survey for up to 18 months. In-home assistance was the most frequently reported type of out-of-pocket care expense and the costliest. care partners who paid for in-home assistance or respite reported more behavioral and psychological symptoms of dementia behaviors, higher reactivity, and higher burden than those who did not.

**Conclusions:**

This novel web-based weekly survey–based approach offers lessons for designing and implementing future cost-focused studies and care partner–supportive telehealth-based interventions for Alzheimer disease and related dementias (ADRD). The results correspond with the existing understanding of ADRD in that high family-related out-of-pocket costs are a typical part of the caregiving experience, and those costs likely increase with dementia severity. The results may also offer potential insights to health systems and policy makers as they seek to implement telehealth-based and related interventions that seek to better support people living with ADRD and their family care partners.

**Trial Registration:**

ClinicalTrials.gov NCT04335110; https://clinicaltrials.gov/ct2/show/NCT04335110

## Introduction

More than 55 million people around the world are living with Alzheimer disease and related dementias (ADRD) [1]. Considered to be the most expensive disease in the United States with annual health and long-term care costs surpassing US $360 billion in 2024, an estimated 6.9 million Americans are living with ADRD [2,3]. An important component of the societal costs of ADRD is the family-level financial impacts associated with caring for an individual living with ADRD, which are often high and driven in part by the prolonged course and intensity of the disease and the heavy care demands placed on family care partners. According to the annual Facts and Figures report by the Alzheimer’s Association, over 11.4 million care partners in the United States are providing more than US $346.5 billion worth of uncompensated care each year [3].

A significant contributor to care demands, and therefore the costs of care, are the behavioral and psychological symptoms of dementia (BPSD; eg, depression, irritability, agitation, and anxiety), which are commonly experienced by individuals living with ADRD [4]. The changing and increasing behaviors as the disease progresses are some of the most challenging aspects of the ADRD journey for family care partners [5,6]. The total lifetime costs of ADRD care are estimated at US $377,621 per individual (in 2021), while as much as 70% of all the lifetime costs of care are carried by family care partners through unpaid care and support, and result in significant out-of-pocket expenses [7,8]. A number of studies have assessed the costs associated with behavioral symptoms and dementia using sources such as health care claims data, physician-reported data, and care partner interviews [9-12]. These approaches all have their unique advantages, but also important limitations for understanding costs. Among these limitations, little is known about the specific relationships between behavioral symptoms, and the out-of-pocket costs borne by family care partners, or how those costs may evolve over time [13].

Support via Technology: Living and Learning with Advancing Alzheimer Disease (STELLA) is a telehealth-based intervention designed to address behavioral symptoms through a personalized approach to teaching family care partners strategies to help manage behavioral symptoms [14-16]. To better understand the potential cost-related impacts of this intervention and the relationship between behavioral symptoms and household ADRD costs, care partners enrolled in the STELLA intervention completed weekly web-based surveys about the out-of-pocket costs associated with care-related activities, care partner time dedicated to those care-related activities, and several physical and mental health–related questions for both the care partner and care recipient (Multimedia Appendix 1).

This report’s goal is to provide needed initial evidence on the costs of caring for a family member with dementia. The primary aim was to determine the feasibility and acceptability of using a novel method to ascertain out-of-pocket costs associated with dementia care: a web-based weekly survey to capture real-time data on caregiving expenses and time commitments completed by care partners. Feasibility was measured by accrual, retention, and data completion. The secondary aims were to examine the relationships between BPSD behaviors and care partner reactivity (as measured by baseline Revised Memory and Behavioral Problems Checklist-Frequency [RMBPC-F] and Revised Memory and Behavioral Problems Checklist-Reaction [RMBPC-R] total scores respectively) with self-reported dementia care costs [17].

We also examined the relationship between care partner burden (as measured by baseline Zarit Burden Index [ZBI] screen total score) and self-reported dementia care costs [18].

A final exploratory aim was to examine changes in burden and costs pre- and post-STELLA telehealth-based intervention (see results in Multimedia Appendix 2).

These data were gathered during the STELLA behavioral intervention and follow-up period (up to 18 months). Basic information on costs was collected prior to and after the intervention. The study used a novel design that provided granular cost data (collected weekly) during a technology-based intervention for ADRD care partners, and insight into the relationship between objective measures of burden in relation to implicit (eg, time) and out-of-pocket costs. Weekly queries of care partners on out-of-pocket costs allowed for more precise measurement of fluctuations in ADRD-induced costs that occur during the trajectory of the disease process that a simple pre-post survey does not provide. These findings may help prepare for effective future scaling of this intervention and help to determine the longer-term financial impacts of implementing this and other ADRD interventions in the community.

## Methods

### Recruitment

STELLA participants were recruited from an existing cohort of participants with ADRD, and their care partners, who were enrolled in the ORCASTRAIT Life Laboratory based at the Oregon Center for Aging & Technology (ORCATECH).

### Intervention

STELLA is a videoconference-based multicomponent intervention designed to facilitate effective management of the upsetting behavioral symptoms that come with dementia progression. In STELLA intervention, professionals (“guides”) meet with family members (“care partners”) for 1 hour/week for 8 weeks to identify strategies to address distressing care–recipient behaviors [14-16]. The goal of STELLA is to reduce upsetting behaviors that are common in the later stages of dementia, and thus care partner burden. We assessed out-of-pocket costs that are incurred by families.

STELLA was developed from the STAR-Caregiver (Staff Training in Assisted Living Residences-Caregivers) program [14]. It was modified to be administered via telehealth [15,16,19]. The cost data reported here were captured as part of a STELLA pilot study (ClinicalTrials.gov, NCT04335110) conducted through the Oregon Roybal Center for Care Support Translational Research Advantaged by Integrating Technology (P30 AG024978-19).

BPSD behaviors and care partner reactivity were assessed at baseline and post-STELLA intervention via the RMBPC-F and RMBPC-R total scores (maximum=96) completed via computerized assessment by care partner [17]. Care partner burden was measured using a 4-item ZBI screen total score (maximum=16) [18].

### Survey Development

To better understand the relationships between behavioral symptoms and out-of-pocket costs, care partners living with a person with ADRD who enrolled in the STELLA intervention completed weekly surveys. Cost-focused questions were developed to be a component of the wider ORCATECH weekly surveys. The surveys were delivered via email to participants every Monday morning by ORCATECH using the Qualtrics survey platform (Qualtrics) using a previously established protocol [20,21]. The weekly surveys included several cost- and time-specific questions, which focused on the out-of-pocket costs care partners incurred from hospitalizations and emergency department (ED) use, primary care providers visits, use of paid in-home care and respite services, prescription medications, and use of over-the-counter (OTC) medications while providing care and support. Questions were designed to be completed in a few minutes or less. To avoid added care partner burden, estimates of costs for care-related items were asked of participants rather than exact figures. Further, cost estimate bands (eg, US $1-$100) rather than asking care partners to recall a specific amount were also used in these cost-related questions to reduce potential burden. See Multimedia Appendix 1 for weekly cost-focused questions and possible responses. The amount of time dedicated to care-related activities was also measured through the weekly surveys by asking care partners how much time they dedicated to these same activities (eg, hospitalizations and ED use, primary care visits, use of paid in-home care, prescription medications, and use of OTC medications).

### Ethical Considerations

The study protocol was reviewed and approved by the Oregon Health & Science University institutional review board (approval #19306). All human participants provided their informed consent to participate. Study consent forms were reviewed and approved by the institutional review board. All due care was taken to protect the privacy and confidentiality of all study participants both during and after the study concluded. All data presented in this work have been deidentified. Therefore, identification of individual participants in this study is not possible. No financial compensation was provided to study participants.

### Statistical Analysis

Summary statistics (mean, SD; or count, %) were generated for care partner and care recipient demographics and baseline clinical measures (dementia severity, presence of behavior problems, depression, and burden scores). The overall prevalence of endorsing any out-of-pocket expenses and the 5 subscale expense questions was calculated over all surveys and by care partner. Interval responses for specific cost and time questions were dichotomized for reporting purposes. Differences in care partner and care recipient characteristics among those who ever endorsed assistance or respite costs (including in-home care) during the study period versus never endorsing these costs were examined using 2-sample independent 2-tailed *t* tests for continuous variables and chi-square test for categorical variables. Differences in pre-post STELLA intervention measures and costs endorsements were examined using paired *t* tests or McNemar chi-square test for matched pairs as appropriate. Due to the small sample size, we were unable to control for covariates. Analyses were performed using SAS software (version 9.4; SAS Institute).

## Results

A total of 13 care partners and their 13 care recipients living with ADRD enrolled in STELLA. Care recipients did not take part in the STELLA intervention but were consented to assure ethical use of their data. Two care partner dyads withdrew after the 8-week STELLA intervention due to worsening health of their care recipients, but weekly survey data were collected during their participation. This pilot cohort was White non-Hispanic and highly educated; care recipients were on average 4 years older than care partners. Care recipient dementia severity ranged from mild to severe. Care partners initially reported relatively high levels of behavioral problems, relatively high reactivity and moderately high burden. In this small group, higher BPSD behaviors and care partner reactivity (RMBPC-F and RMBPC-R total scores) were marginally or significantly correlated with higher care partner burden (ZBI total score) and depression (Center for Epidemiologic Studies Depression Scale total score) [17,18,22]. Characteristics of STELLA study participants are presented in Table 1.

**Table 1:** STELLA^a^ intervention participant characteristics.

Variable			Care partner (n=13)		Care recipient (n=13)
Age (years), mean (SD)			72.8 (8.0)		76.6 (9.8)
Sex (female), n (%)			9 (69)		5 (36)
Education (years), mean (SD)			16.3 (1.9)		15.5 (2.5)
Race (White non-Hispanic), n (%)			13 (100)		13 (100)
Years spent caregiving, mean (SD)			4.4 (2.2)		N/A^b^
CDR-SOB^c^, mean (SD)			N/A		10.0 (4.2)
**Dementia severity, n (%)**					
	MCI^d^ or mild AD^e^	N/A		5 (38)	
	Moderate AD	N/A		6 (46)	
	Severe AD	N/A		2 (15)	
RMBPC-F^f^ total score, mean (SD)			41.5 (12.0)		N/A
RMBPC-R^g^ total score, mean (SD)			27.5 (14.9)		N/A
CESD^h^ total score, mean (SD)			9.9 (7.2)		N/A
ZBI^i^ total score, mean (SD)			7.7 (2.8)		N/A

^a^STELLA: Support via Technology: Living and Learning with Advancing Alzheimer Disease.

^b^N/A: data are not applicable or not available.

^c^CDR-SOB: Clinical Dementia Rating Scale-Sum of Boxes Scores.

^d^MCI: mild cognitive impairment.

^e^AD: Alzheimer disease.

^f^RMBPC-F: Revised Memory and Behavior Problems Checklist-Frequency.

^g^RMBPC-R: Revised Memory and Behavior Problems Checklist-Reaction.

^h^CESD: Center for Epidemiologic Studies Depression Scale.

^i^ZBI: Zarit Burden Interview.

### Feasibility and Acceptability

During the 8-week STELLA behavioral intervention and follow-up period (up to 18 months) 486 weekly cost surveys were completed by the 13 care partners for a mean of 37 cost surveys per care partner (SD 24; range 3-81 forms). On average, care partners completed 66% (SD 28%; range 29%-100%) of all weekly surveys sent to them. The mean intervention study time during which forms were completed was 229 days (SD 127; range 7-406 days). Generally, care partners found the weekly cost questions acceptable and easy to complete and not time-consuming. No technical issues were encountered with the surveys. The average time to complete the costs questions was under 10 seconds (about 30 seconds if any expenses were endorsed). Many were motivated to participate in research to help other families in the future.

### Types and Prevalence of Out-of-Pocket Dementia Care Costs

Over the study period, nearly all care partners (n=12, 92%) ever reported any dementia care–related expenses not paid for by health insurance (eg, Medicare, Medicaid, or private insurance), and most care partners reported out-of-pocket costs related to primary care visits, respite care, prescription meds and OTC items (Table 2). Nearly half (n=214, 44%) of all weekly surveys endorsed some caregiving expenses. The most frequently endorsed out-of-pocket dementia care expense (and the costliest) across all weekly surveys collected was assistance or respite care (n=164, 77.6% of all surveys with any expenses). We subsequently focused our interest on the prevalence of assistance or respite care costs. Of all surveys with dementia care expenses, 94 (44%) paid for prescription drugs, 71 (33%) paid for OTC items, 41 (19%) paid for primary care visits, and 10 (5%) paid for ED or hospital visits.

**Table 2:** Types of out-of-pocket dementia care–related expenses reported during the study period by care partners (n=13).

	
Expense Variable	Values, n (%)
Expenses (any)	12 (92)
Emergency department or hospitalization-related	5 (38)
Primary care–related	9 (69)
Prescription medications	11 (85)
In-home assistance or respite care	8 (62)
Over-the-counter medications or care items	9 (69)

### Specific Costs: Expenses and Time

Care partners were asked to report costs and time for specific types of dementia care expenses by choosing one of several “brackets” from US $1 to greater than US $1000 and from less than 15 minutes to greater than 5 hours respectively. When in-home assistance or respite care costs were reported, the vast majority (n=149, 90%) paid more than US $100 per week while 49 (30%) reported paying more than US $500 per week. For ED or hospital visits (n=10), 3 reported paying more than US $1000 out-of-pocket; and five reported spending more than 5 hours assisting with the visit (travel time and wait time). When prescription drug costs were reported, most paid less than US $100 but a subset (n=15, 16%) paid more than US $100 on copays. When OTC medication costs were reported, most (n=60, 86%) paid less than US $100 per week. For primary care visits, a large subset (n=18, 44%) paid more than US $100 out-of-pocket for these visits, and 63% (n=5) of respondents spent 1 hour or more assisting with the visit. The overall frequency of reported weekly costs categorized by dollar amount for dementia-related in-home assistance or respite care are presented in Table 3.

**Table 3:** Overall frequency of reported weekly costs categorized by dollar amount for dementia-related in-home assistance or respite care.

Amount (US $)	Frequency, n
1-100	15
101-200	55
201-300	16
301-400	20
401-500	9
501-600	38
601-700	5
701-800	3
801-900	2
901-1000	0
≥1001	1

### Associations Between Out-of-Pocket Costs for Assistance or Respite Care and Care Partner or Care Recipient Characteristics

To investigate the relationship between behavioral symptoms and costs we examined group differences between care partners who did and did not pay for in-home assistance or respite care during the study (Table 4). Of 13 care partners, 8 (62%) endorsed paying for assistance or respite care and 5 (38%) did not. Care partners who paid for some assistance or respite care were relatively younger and more highly educated than those who did not. Care partners who paid for some assistance or respite care had care recipients with higher dementia severity as measured by Clinical Dementia Rating Scale-Sum of Boxes Scores. BPSD behaviors and care partner reactivity as measured by RMBPC-F and RMBPC-R total scores were relatively higher among those who paid for assistance or respite care [17]. Caregiver burden as measured by the 4-item ZBI score was also relatively higher among those who paid for assistance or respite care [18]. Even with a very small sample size, the results suggest that the frequency of BPSD behaviors, care partner reactivity, and burden are associated with deciding to pay for in-home assistance or respite care. The effect sizes for RMBPC-F and ZBI scores as measured by Cohen *d* are considered large (*d*=0.8).

**Table 4:** Baseline participant characteristics among care partners who did and did not pay for in-home assistance or respite care during STELLA^a^.

Variable	Never endorsed paying for assistance or respite care (n=5)	Endorsed paying for assistance or respite care (n=8)	*P* value
Care partner age (years), mean (SD)	76.8 (8.2)	69.5 (6.6)	.10
Care partner sex (female), n (%)	4 (80)	5 (63)	≥.99
Care partner education, years, mean (SD)	14.6 (2.4)	16.8 (1.5)	.07
Years spent caregiving, mean (SD)	3.8 (1.5)	4.8 (2.6)	.48
Care recipient age (years), mean (SD)	81.2 (10.3)	73.8 (9.0)	.19
Care recipient CDR-SOB^b^, mean (SD)	7.7 (3.5)	11.5 (4.2)	.12
RMBPC-F^c^ total score, mean (SD)	34.4 (11.0)	46.0 (10.8)	.09
RMBPC-R^d^ total score, mean (SD)	24.6 (10.8)	29.3 (17.5)	.61
CESD^e^ total score, mean (SD)	7.2 (7.2)	11.6 (7.1)	.30
ZBI^f^ total score, mean (SD)	6 (2)	8.8 (2.9)	.09

^a^STELLA: Support via Technology: Living and Learning with Advancing Alzheimer Disease.

^b^CDR-SOB: Clinical Dementia Rating Scale-Sum of Boxes Scores.

^c^RMBPC-F: Revised Memory and Behavior Problems Checklist-Frequency.

^d^RMBPC-R: Revised Memory and Behavior Problems Checklist-Reaction.

^e^CESD: Center for Epidemiologic Studies Depression Scale.

^f^ZBI: Zarit Burden Interview.

### Comparison of Burden Measures and Costs Before and After the STELLA Intervention

There were no significant differences found on burden measures or costs before and after the STELLA pilot intervention in this small cohort (Multimedia Appendices 2 and 3).

## Discussion

### Principal Findings

The results of this study suggest weekly web-based surveys focused on out-of-pocket expenses and time associated with care and support-related activities for someone living with ADRD are a possible approach to a more direct measurement method for the longitudinal capture of financial and time costs of caregiving. Overall, participating care partners (n=13) found the weekly cost questions to be acceptable, easy to complete, and not time-consuming. The average time to complete the survey costs questions was less than 10 seconds or approximately 30 seconds if any care-related expenses were endorsed. Our team found high motivation to participate in the study based on a desire to help others experiencing the dementia caregiving journey. During the recruitment and onboarding process, a few participants expressed reticence toward reporting personal financial information related to dementia care. However, this reticence was easily mitigated by discussion of the expense-related questions and further explanation of the purpose of collecting and analyzing these data. On average, each care partner completed two-thirds of all weekly surveys sent to them with moderate variability (mean 66%, SD 28%; range 29%-100%) suggesting the feasibility of this novel method for collecting the financial and time costs of caregiving.

Yet, some of the care partners in our study did not complete all weekly surveys, and some only completed a few, suggesting that the surveys may need some revision to ensure they can fit into the multiple demands on care partner time. In a follow-up study, a more formal usability acceptance analysis is warranted. In addition, some type of post-participation stipend (eg, gift card) may also help with survey adherence. In Table 2, 92% (n=12) of study participants ever endorsed some level of out-of-pocket care expenses during the monitoring period, confirming that out-of-pocket expenses are a common part of the experience of caring for someone living with ADRD as other previous studies have similarly demonstrated [23-25]. Most care partners (n=11, 85%) ever endorsed paying for prescription medications and 69% (n=9) ever endorsed paying for OTC medications or care items, while 62% (n=8) of care partners ever endorsed paying for assistance or respite care. This also aligns with previous ADRD cost-focused studies, which show that the largest share of annual ADRD out-of-pocket costs is incurred by paying for medications and in-home care assistance [25]. Using weekly cost queries, we showed that this cost burden can be observed within a period of less than a year.

Not only are ADRD care–related activities costly for care partners, but also they are often highly time-consuming and increase in duration along with disease progression [8,26]. Moreover, previous studies have demonstrated that more care partner time devoted to caring is associated with higher rates of care partner depression and other poor health outcomes [27]. In terms of our measurement of care partner time dedicated to care-related activities, that 70% (7/10) of care partner participants in our study spent 3 or more hours assisting with a visit to an ED or hospitalization for their care recipient is not surprising given these events are often complex, highly disruptive, and time-consuming events. Furthermore, existing research shows that there is an increased rate of hospitalization among people living with ADRD compared to people without ADRD, while the number of potentially avoidable hospitalizations of people living with ADRD is also increasing [28,29]. This finding reinforces the argument for better interventions that are able to reduce the need for ED visits and hospitalizations among persons living with ADRD. Further, in terms of weekly reports on the amount of time devoted to primary care provider visits, 63% (n=5) of respondents spent 1 hour or more assisting with the visit. This also aligns with existing expectations given that health care visits for a person living with ADRD are often complex, disruptive, and take a lot of care partner (and health care provider) time in preparation and in attendance [30]. Of all reports on OTC medications, 52% (37/71) of care partners spent 1 hour or more on this activity. While providing assistance with medications may be a routine part of the caregiving experience, these findings demonstrate that considerable time is dedicated to this activity on an ongoing basis. Interventions that can help to minimize the amount of time dedicated to this activity may be helpful, such as medication training for care partners.

Of the 13 care partners who participated in STELLA, 8 (62%) ever endorsed paying for assistance or respite care during the study period and 5 (38%) did not. We found no differences between these 2 groups by baseline care recipient or care partner age, or years spent caregiving. Care partners who paid for some assistance or respite care were relatively younger and more highly educated than those who did not. Care partners who paid for some assistance or respite care had care recipients with higher dementia severity as measured by Clinical Dementia Rating Scale-Sum of Boxes Scores. BPSD behaviors and care partner reactivity, as measured by RMBPC-F and RMBPC-R total scores, were relatively higher among those who paid for assistance or respite care [12,13,17]. Care partner burden as measured by the 4-item ZBI score was also relatively higher among those who paid for assistance or respite care [18].

Despite the small sample size, the results of this study suggest that the frequency of BPSD behaviors, care partner reactivity, and care partner burden are associated with the decision of families to pay for assistance or respite care. This in turn suggests that telehealth-based interventions such as STELLA may lower the out-of-pocket costs experienced by families when dementia caregiving, specifically in-home assistance and respite care costs, which are particularly burdensome for families and have limited coverage under existing programs or insurance [25]. While new programs such as Medicare’s Guiding an Improved Dementia Experience Model seek to provide dementia care partners with much needed support, the out-of-pocket costs of dementia care are likely to remain high for many families [31,32]. Continued use and evaluation of interventions that can reduce the out-of-pocket costs of dementia is needed.

### Limitations

There were several limitations of this study. One limitation is the small sample of family care partners (n=13). Therefore, any comparisons made based on the reported *P* values should be made with caution. A roller-coaster of events related to the SARS-CoV-2 (COVID-19) pandemic greatly affected participant enrollment in this study, a common challenge faced by many clinical trials during this time frame including those in ADRD research [33-35]. These pandemic-induced challenges included health system restrictions regarding being seen or assessed in home or in clinic settings (although the STELLA intervention and web-based queries were all delivered remotely) as well as when relaxed, contact requirements became available potential study participants remained hesitant to engage in research [36]. Reduced mobility, increased telehealth usage, and reductions in in-person caregiving during the COVID-19 pandemic could also have affected this study’s results [37-39]. The results of this study are thus to be interpreted in the context that some of the expenses being reported were incurred under pandemic conditions. Future studies will need to examine this methodology, and the data collected under different conditions and with larger numbers of participants.

Further, all study participants were based within a small geographic area: Oregon, United States. Sociodemographic characteristics of participants including race, ethnicity, and education were also highly homogenous as all 13 care partners and their care recipients were White, non-Hispanic, and not representative of the wider population of older adults in the United States. Using a digital survey may have resulted in a more digitally connected and internet savvy sample of older adults therefore contributing to a homogenous sample [40]. Taken together, these limitations make the generalizability of these results beyond the regional context difficult. A larger study, one that uses weekly surveys of ADRD care partners engaged in this type of intervention that can assess feasibility across diverse populations is needed. The data collected in this study can help in the design of that larger study. Indeed, the results from this study may help prepare for effective future scaling of the STELLA intervention as well as the efficacy of this intervention [41]. As this study shows that the collection of weekly cost data through a web-based survey-based approach is possible, future studies could implement this approach to data collection, demonstrating how costs may shift over time and when costs may increase or decrease based on a broad set of care and support needs of people living with ADRD. In addition, this methodology could readily be implemented in all types of intervention studies, including pharmacologic interventions, to determine the cost efficacy of an intervention in potentially reducing the expenditures and effort of care.

Another limitation of this study was the collection of cost data through the use of cost estimate brackets (ranges). While this approach was intended to reduce the potential burden on participating care partners so they would not have to recall exact figures, we were unable to capture exact costs of care. A further limitation of this study is that it did not collect data on income or the insurance status of participants. Collecting income data would help to elucidate the financial impacts out-of-pocket dementia care costs have on families across socioeconomic groups and the decision to seek paid support. Further, while it is likely that Medicare is the primary source of health coverage of study participants due to an average participant age of 77 years, Medicaid status (dual eligible) or whether they received Veterans Administration (VA) services is not clear. While some coverage of in-home care and respite is covered through Medicaid and the VA (ie, VA Caregiver Support Program), both have strict eligibility requirements. A more detailed understanding of gaps in coverage and out-of-pocket care costs is important for future program and policy development to better support dementia care partners and care recipients.

### Conclusions

A longitudinal weekly survey-based approach to quantifying care partner out-of-pocket costs and care partner time dedicated to care activities is a novel approach to assessing real-world costs related to caring for someone living with ADRD. The preliminary findings of our study correspond with the existing literature and general understanding of ADRD that high family-related out-of-pocket costs are a typical part of the caregiving experience, and those costs likely increase with dementia severity. Both the challenges and benefits of this survey-based approach can offer lessons for designing and implementing future ADRD cost–focused studies. The results may also offer potential insights to health systems and policy makers as they seek to implement telehealth-based and related interventions that seek to better support people living with ADRD and their family care partners.

## Appendix

Multimedia Appendix 1 Weekly survey cost-focused questions.

Multimedia Appendix 2 STELLA (Support via Technology: Living and Learning with Advancing Alzheimer Disease) pilot intervention efficacy results.

Multimedia Appendix 3 Prevalence (count) of types of care expenses during weeks before an after the STELLA (Support via Technology: Living and Learning with Advancing Alzheimer Disease) pilot intervention.

## Abbreviations

ADRD: Alzheimer disease and related dementias
BPSD: behavioral and psychological symptoms of dementia
ED: emergency department
ORCATECH: Oregon Center for Aging & Technology
OTC: over-the-counter
RMBPC-F: Revised Memory and Behavior Problems Checklist-Frequency
RMBPC-R: Revised Memory and Behavior Problems Checklist-Reaction
STAR-Caregiver: Staff Training in Assisted Living Residences-Caregivers
STELLA: Support via Technology: Living and Learning with Advancing Alzheimer Disease
VA: Veterans Administration
ZBI: Zarit Burden Interview

## Data Availability

The data sets generated during and analyzed during this study are available from the corresponding author on reasonable request.
