# Supplementary material for: Ascertaining Out-of-Pocket Costs of Dementia Care: Feasibility Study of a Web-Based Weekly Survey
Source: JMIR Form Res. 2024 Sep 25;8:e56878. doi: 10.2196/56878 (PMC11464940; doi:10.2196/56878)
Supplement: Multimedia Appendix 1 [file formative_v8i1e56878_app1.docx]

| 1. **Any Expenses**   In the last week, did you pay for any care expenses for your family member with dementia that was not paid for by insurance? This could include urgent care visits, medications, adult diapers, co-pays for doctor visits, adult day programs, and other costs not listed.  Choice: 1': 'Yes', '2': 'No'   1. **Emergency Department Visit / Hospital / Urgent Care Visit**   Did your family member with dementia receive urgent care, hospital or emergency department care?  Choice: 1': 'Yes', '2': 'No'   1. **Emergency Department / Hospital / Urgent Care Expenses**   In the last week, how much have you paid out-of-pocket, in dollars, for their urgent care and/or hospital or emergency room visit(s)? Cost examples include co- pays, co-insurance or ambulance services not covered by insurance. - Selected Choice  '0': 'I did not pay anything', '1': '$1 to $100', '2': '$101 to $200', '3': '$201 to $300', '4': '$301 to $400', '5': '$401 to $500', '6': '$501 to $600', '7': '$601 to $700', '8': '$701 to $800', '9': '$801 to $900', '10': '$901 to $1,000', '11': '$1,001 or above (specify).   1. **Emergency Department / Hospital / Urgent Care Time**   How much time did you spend assisting with their visit at urgent care and/or hospital or emergency room? Time examples include travel time, waiting time.  '1': 'I did not assist', '2': '&lt;15 min', '3': '16 to 30 min', '4': '31 to 45 min', '5': '46 min to 1 hour', '6': '1 to 2 hours', '7': '3 to 4 hours', '8': '5 or more hours'   1. **Primary Care Provider Visit**   Did you pay for any costs related to visits with your family member’s primary care provider (doctor, nurse practitioner, physician’s assistant)? Examples include cost of travel to the provider, co-pays, or co-insurance.  Choice: 1': 'Yes', '2': 'No'   1. **Primary Care Provider Expenses**   In the last week, how much have you paid out-of-pocket, in dollars, for items relating to your family member's primary care provider? Cost examples include co- pays, co-insurance, lab fees” anything not covered by insurance. - Selected Choice  ‘0': 'I did not pay anything', '1': '$1 to $100', '2': '$101 to $200', '3': '$201 to $300', '4': '$301 to $400', '5': '$401 to $500', '6': '$501 to $600', '7': '$601 to $700', '8': '$701 to $800', '9': '$801 to $900', '10': '$901 to $1,000', '11': '$1,001 or above (specify).   1. **Primary Care Provider Time**   How much time did you spend assisting with their primary care doctor visit? Time examples include travel time, waiting time.  '1': 'I did not assist', '2': '<15 min', '3': '16 to 30 min', '4': '31 to 45 min', '5': '46 min to 1 hour', '6': '1 to 2 hours', '7': '3 to 4 hours', '8': '5 or more hours’   1. **Prescription Drug Use**   Did you pay for any prescription drugs for your family member with dementia?  Choice: 1': 'Yes', '2': 'No'   1. **Prescription Drug Cost**   In the last week, how much have you paid out-of-pocket, in dollars, for your family member's prescription drugs? Cost examples include co-pays, co-insurance, mail costs. - Selected Choice  '0': 'I did not pay anything', '1': '$1 to $100', '2': '$101 to $200', '3': '$201 to $300', '4': '$301 to $400', '5': '$401 to $500', '6': '$501 to $600', '7': '$601 to $700', '8': '$701 to $800', '9': '$801 to $900', '10': '$901 to $1,000', '11': '$1,001 or above (specify)'   1. **Prescription Drug Time**   How much time did you spend assisting with their prescription drugs? Time examples include travel time, shopping time, preparing, and/or administering.  Choice: '1': 'I did not assist', '2': '<15 min', '3': '16 to 30 min', '4': '31 to 45 min', '5': '46 min to 1 hour', '6': '1 to 2 hours', '7': '3 to 4 hours', '8': '5 or more hours'   1. **Assistance or Respite Use**   Did you pay for any assistance or respite care? Examples include payments made to an adult day program or for paid in-home caregivers.  Choice: 1': 'Yes', '2': 'No'   1. **Assistance or Respite Expenses**   In the last week, how much have you paid out-of-pocket, in dollars, for assistance or respite care? Cost examples include fees for paid caregivers. - Selected Choice  '0': 'I did not pay anything', '1': '$1 to $100', '2': '$101 to $200', '3': '$201 to $300', '4': '$301 to $400', '5': '$401 to $500', '6': '$501 to $600', '7': '$601 to $700', '8': '$701 to $800', '9': '$801 to $900', '10': '$901 to $1,000', '11': '$1,001 or above (specify)'   1. **Assistance or Respite Time**   How much time did you spend on respite care or assistance? Time examples include time spent on interviews of caregivers.  '1': 'I did not assist', '2': '<15 min', '3': '16 to 30 min', '4': '31 to 45 min', '5': '46 min to 1 hour', '6': '1 to 2 hours', '7': '3 to 4 hours', '8': '5 or more hours'   1. **Over the Counter Drugs Use**   In the last week, did you pay for non- prescriptions drugs (such as Tylenol, ibuprofen), or personal care items for your family member with dementia?  Choice: 1': 'Yes', '2': 'No'   1. **Over the Counter Drugs Expenses**   In the last week, how much have you paid out-of-pocket, in dollars, for non- prescription drugs or personal care items for your family member? Cost examples include adult diapers, therapeutic creams, such as Destin, or bandages. Please provide an estimate on dollar amount. - Selected Choice  '0': 'I did not pay anything', '1': '$1 to $100', '2': '$101 to $200', '3': '$201 to $300', '4': '$301 to $400', '5': '$401 to $500', '6': '$501 to $600', '7': '$601 to $700', '8': '$701 to $800', '9': '$801 to $900', '10': '$901 to $1,000', '11': '$1,001 or above (specify)'   1. **Over the Counter Drugs Time**   How much time did you spend assisting with non-prescription drugs or personal care items for your family member? Time examples include travel time, shopping time, preparing, and/or administering.  '1': 'I did not assist', '2': '<15 min', '3': '16 to 30 min', '4': '31 to 45 min', '5': '46 min to 1 hour', '6': '1 to 2 hours', '7': '3 to 4 hours', '8': '5 or more hours' |
| --- |
|  |
