# Supplementary material for: Ascertaining Out-of-Pocket Costs of Dementia Care: Feasibility Study of a Web-Based Weekly Survey
Source: JMIR Form Res. 2024 Sep 25;8:e56878. doi: 10.2196/56878 (PMC11464940; doi:10.2196/56878)
Supplement: Multimedia Appendix 2 [file formative_v8i1e56878_app2.docx]

|  | **Pre-STELLA** | **Post-STELLA** | **p-value** ^b^ |
| --- | --- | --- | --- |
|  | n=13 ^a^ | n=13 ^a^ |  |
| **Variable** |  |  |  |
| RMBPC-F total score | 41.5 (12.0) | 39.5 (13.9) | 0.35 |
| RMBPC-R total score | 27.5 (14.9) | 24.7 (13.0) | 0.24 |
| CESD total score | 9.9 (7.2) | 9.5 (6.7) | 0.47 |
| ZBI total score | 7.7 (2.8) | 7.9 (2.4) | 0.61 |
| QOL Caregiver total score | 37.1 (5.3) | 35.5 (6.2) | 0.23 |
| QOL PWD total score | 33.3 (7.7) | 34.0 (7.8) | 0.62 |

^a^ Mean (standard deviation) or percentage as appropriate.

^b^ Paired t-test or McNemar's chi-square test for matched pairs.
