# Supplementary figures and images for: Ascertaining Out-of-Pocket Costs of Dementia Care: Feasibility Study of a Web-Based Weekly Survey
Source: JMIR Form Res. 2024 Sep 25;8:e56878. doi: 10.2196/56878 (PMC11464940; doi:10.2196/56878)

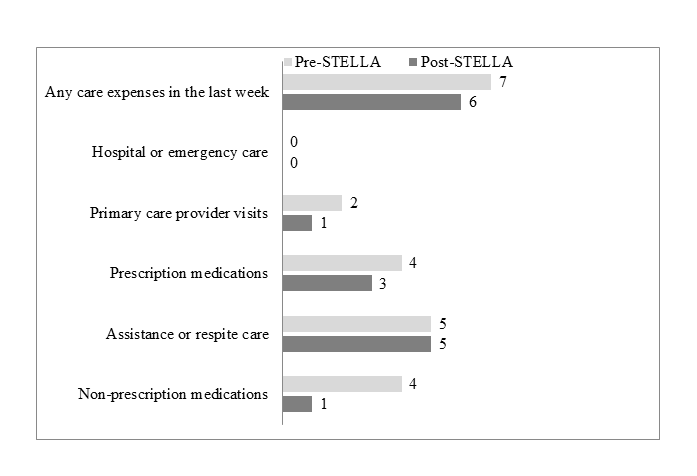

Supplement: Multimedia Appendix 3 [file formative_v8i1e56878_app3.png]
